# Oral administration of the probiotic strain *Lactobacillus helveticus* BGRA43 reduces high-fat diet–induced hepatic steatosis in mice and restores gut microbiota composition

**DOI:** 10.3389/fphar.2025.1688777

**Published:** 2025-11-04

**Authors:** Ana Teofilović, Miloš Vratarić, Biljana Bursać, Ljupka Gligorovska, Danijela Vojnović Milutinović, Nemanja Stanisavljević, Ivana Strahinić, Danijela Mišić, Filip Nikolić, Bojan Pavlović, Katarina Jončić Savić, Cem Aydogan, Ana Djordjevic

**Affiliations:** ^1^ Department of Biochemistry, Institute for Biological Research “Siniša Stanković” - National Institute of the Republic of Serbia, University of Belgrade, Belgrade, Serbia; ^2^ Department for Microbiology and Plant Biology, Institute of Molecular Genetics and Genetic Engineering, University of Belgrade, Belgrade, Serbia; ^3^ Department of Plant Physiology, Institute for Biological Research “Siniša Stanković” - National Institute of the Republic of Serbia, University of Belgrade, Belgrade, Serbia; ^4^ Phytonet DOO, Belgrade, Serbia; ^5^ Phytonet AG, Schindellegi-Feusisberg, Switzerland

**Keywords:** liver, steatosis, probiotics, *Lactobacillus* helveticus BGRA43, gut microbiota, lipid metabolism, inflammation

## Abstract

**Introduction:**

The prevalence of metabolic dysfunction-associated steatotic liver disease (MASLD) is rapidly increasing. Modulation of the gut microbiota through the use of probiotics has been recognized as an important option for the treatment of hepatic steatosis. Previous studies suggested that the bacterial strain *Lactobacillus helveticus* BGRA43 (LHBGRA43) can reduce inflammation and improve the bacterial balance in the gut. The aim of this study was to investigate whether oral administration of LHBGRA43 in mice fed a high-fat diet contributes to the reduction of hepatic steatosis through its beneficial effects on the composition of the gut microbiota.

**Methods:**

Male C57BL/6J mice (2.5 months old) were divided into three groups: a control group fed a standard diet (10% kcal fat), a high-fat diet (HFD) group (60% kcal fat for 14 weeks) and a HFD group that received freeze-dried LHBGRA43 dissolved in PBS orally for the last 5 weeks of the diet.

**Results:**

Histological analysis of the liver showed that animals fed HFD exhibited hepatic steatosis, while no lipid droplets were present in the liver of animals receiving LHBGRA43. This decrease in steatosis correlated with decreased level of sterol regulatory element-binding protein-1c, reduced expression of the fatty acid transporter *Cd*36, enzymes involved in ceramide synthesis and proinflammatory markers. The administration of LHBGRA43 also improved the integrity of the small intestine barrier, as evidenced by an increased level of ZO-1 protein. The observed reduction in intestinal permeability was associated with a decreased *Firmicutes/Bacteroidota* ratio and increased abundance of the genera *Alistipes, Acetatifactor* and *Odoribacter*, as well as a decreased concentration of branched-chain 4-methylvaleric acids.

**Discussion:**

In conclusion, the restoration of the gut microbiota composition in combination with the strengthening of the small intestine barrier suggests that LHBGRA43 could be used as a general probiotic strain with ameliorative effects on hepatic lipid accumulation and lipotoxicity.

## 1 Introduction

Modern lifestyle characterized with high-calorie diets and a lack of physical activity contributes significantly to the global increase in obesity and associated metabolic diseases (Moschonis and Trakman, 2023). The estimated global prevalence of metabolic dysfunction-associated steatotic liver disease (MASLD), formerly known as non-alcoholic fatty liver disease (NAFLD), is approximately 32% of the adult population and continues to increase (Bourganou et al., 2025). MASLD arises from a disturbed balance between hepatic fatty acid synthesis and uptake versus oxidation and release. The disease encompasses a spectrum of liver conditions ranging from simple steatosis to inflammation, fibrosis, and, ultimately, irreversible outcomes such as cirrhosis or hepatocellular carcinoma (Heeren and Scheja, 2021; Bashir et al., 2022).

Hepatic triglyceride accumulation in MASLD occurs predominantly through *de novo* lipogenesis, particularly under conditions of insulin resistance (Carli et al., 2024). This process is stimulated by carbohydrate-rich diets, which enhance the expression and activity of key lipogenic enzymes, including acetyl-CoA carboxylase (ACC) and fatty acid synthase (FAS) (Sanders and Griffin, 2016). If the diet is enriched with fats, the depots of white adipose tissue expand, while the hepatic uptake of free fatty acids (FFA) from the bloodstream *via* the fatty acid translocase (FAT/CD36) is promoted (Mallick et al., 2024). Regardless of the fatty acids source, stearoyl-CoA desaturase 1 (SCD1) facilitates their desaturation, thereby preparing them for esterification (Jeyakumar and Vajreswari, 2022). The resulting monounsaturated fatty acids serve as substrates for acyltransferases, of which diglyceride acyltransferase 1 (DGAT1) plays the most important role as it catalyzes the final step of triglyceride synthesis. When triglycerides are assembled with the perilipin family of proteins, neutral lipid droplets are formed and protected from hydrolysis leading to their accumulation and the development of steatosis (Yen et al., 2008; Sztalryd and Brasaemle, 2017). In contrast, when triglycerides are combined with apolipoproteins under the action of the microsomal triglyceride transfer protein (MTTP), very low density lipoproteins (VLDL) are prepared for release from the liver into the bloodstream (Shindo et al., 2010; Chatrath et al., 2012). In this way, the risk of hepatic steatosis is reduced, but the ectopic accumulation of lipids and dyslipidemia could be provoked. Therapeutic strategies targeting enzymes involved in *de novo* lipogenesis and downstream lipid metabolism can decrease hepatic fat accumulation and slow MASLD progression (Devasia et al., 2025). However, reducing triglyceride and lipid droplet formation while maintaining high hepatic influx of exogenous fatty acids can lead to ceramide and other reactive lipid intermediate accumulation, driving severe steatosis and hepatocellular lipotoxicity (Delcheva et al., 2024). Several studies have demonstrated that inhibition or depletion of ceramide synthase (CERS) and sphingolipid desaturase (DEGS) prevents the development of hepatic steatosis, reduces inflammation and limits cellular stress (Raichur, 2020; Tanase et al., 2021; Zhu et al., 2023).

One of the most promising approaches for treating fatty liver is the use of natural products, as lifestyle interventions such as dietary modification and increased physical activity are often difficult to sustain, and pharmacological agents can cause significant side effects (Kosmalski et al., 2023; Li Y. et al., 2023). Modulation of the gut microbiota through probiotics has recently been recognized as an important mechanism for ameliorating liver disease. Several studies suggest that probiotic strains, particularly *Lactobacillus* spp. and *Bifidobacterium* spp., can enhance gut barrier integrity, reduce inflammation, exhibit antidiabetic properties and positively influence liver function in MASLD (Wang G. et al., 2020; Jeong et al., 2022; Ghosh et al., 2024a; 2024b; Maftei et al., 2024; Oudat and Okour, 2025). *Lactobacillus helveticus* BGRA43 (LHBGRA43), originally isolated from the human gastrointestinal tract, has demonstrated antimicrobial activity against *Yersinia enterocolitica*, *Shigella sonnei*, *Shigella flekneri* and *Streptococcus pneumoniae*, supporting its potential to alleviate gastrointestinal disorders and modulate gut microbiota composition. Furthermore, bioactive peptides released by LHBGRA43 during milk fermentation have been shown to regulate innate immunity by modulating the production of proinflammatory cytokines such as interleukin-6 (IL-6) and tumor necrosis factor alpha (TNF-α) (Strahinic et al., 2013; Strahinić et al., 2013; Lukić et al., 2016).

Previous research on LHBGRA43 has been limited to its antimicrobial and immunomodulatory properties. No studies to date have examined whether this strain can influence hepatic lipid accumulation or directly ameliorate high-fat diet-induced liver steatosis. Additionally, the relationship between LHBGRA43-mediated modulation of gut microbiota and systemic metabolic outcomes remains unexplored. Considering these gaps and the previously reported effects on bacterial balance in the gut, we hypothesized that oral administration of LHBGRA43 reduces hepatic steatosis in high-fat diet-fed mice by beneficially modulating gut microbiota composition. The aim of this study was to determine whether LHBGRA43 can be considered a candidate probiotic for MASLD management. To this end, we evaluated the effects of a 5-week oral administration of LHBGRA43 on physiological and biochemical parameters, liver histology, hepatic lipid metabolism, inflammation, intestinal microbiota composition, bacterial metabolites, and small intestinal barrier integrity in mice fed a 60% high-fat diet.

## 2 Materials and methods

### 2.1 Bacterial preparation

LHBGRA43 isolated from the human gastrointestinal tract was used in the experiment. The bacterial strain LHBGRA43 has been deposited in the Belgian Coordinated Collection of Microorganisms (BCCM), Ghent, Belgium under the accession number LMG P-24226 and is the property of the Institute for Molecular Genetics and Genetic Engineering, University of Belgrade, Serbia (Lozo et al., 2011). For oral gavage of experimental animals, the bacteria were applied in freeze-dried form, prepared according to the manufacturer's specification (Lactosan GmbH and Co. KG, under the batch number M-20-021c, production date 19.02.2020). Freeze dried preparation had specified concentration of 2 × 10^9^ CFU/g, which was verified by serial dilution in physiological solution and plating on DeMan, Rogosa and Sharpe agar (MRS, Merck, Darmstadt, Germany). Plates were incubated at 37 °C under anaerobic conditions (Anaerocult A, Merck, Darmstadt, Germany) for 48 h and viable cell count was determined (data not shown).

### 2.2 Animals and experimental groups

Male 2.5-month-old C57BL/6J mice were randomly selected and housed in pairs in cages with perforated plexiglass which provided physical separation to measure the food intake and collect feces of each animal while enabling communication and reducing social stress isolation. Male mice were selected for the experiment due to their greater susceptibility to obesity and diet-induced steatosis, as well as reduced hormonal variability compared to females. The animals were kept under standard conditions (22 , 12-h light/dark cycle, constant humidity) and had *ad libitum* access to water, with constant veterinary care.

The mice were divided into three experimental groups (n = 8 animals per group). The control group (C) had *ad libitum* access to a commercial diet containing 10 kcal% fat (Rodent Diet with 10 kcal% Fat, D12450J, Research Diets, New Brunswick, USA). The high-fat diet (HFD) group had *ad libitum* access to a 60 kcal% fat diet (Rodent Diet with 60 kcal% Fat, D12492, Research Diets, New Brunswick, USA) during the 14-week treatment period, which has previously been shown to induce hepatic steatosis (Zhou et al., 2020). This diet consists of 60% fat, 20% protein, and 20% carbohydrates, and was designed to induce obesity, insulin resistance, and hepatic steatosis in C57BL/6J mice (https://info.taconic.com/hs-fs/hub/355513/file-2452871853-pdf/Technical_Library/D12492.pdf). The third group, designated as HFD + B, had *ad libitum* access to a high-fat diet for 14 weeks and received 100 µL of freeze-dried LHBGRA43 suspended in PBS via oral gavage for the final 5 weeks of the high-fat diet regimen. A freshly prepared bacterial suspension was used in each treatment. During this period, mice in the HFD + B group were daily administered with 2 × 10^8^ CFU of LHBGRA43. The concentration of bacteria in the treatment was selected based on previous use of this strain in prevention and/or treatment of intestinal infections in mammals (humans and farm animals) and as an immunomodulator (WO/2017/105,267) (Golić et al., 2017). The C and HFD groups received an equivalent daily volume of PBS without LHBGRA43 also by oral gavage. The food intake per animal was measured daily, and daily caloric intake was calculated as follows: for the control group [mass of food consumed per day (g) × 3.85 kcal/g], for the mice on the high-fat diet [mass of food consumed per day (g) × 5.24 kcal/g]. Body mass was measured weekly.

### 2.3 Animal euthanasia and experimental sample collection

At the end of the treatment, the mice were fasted for 4 hours and euthanized by rapid decapitation, performed by a trained veterinarian using a rodent guillotine for humane euthanasia of small animals (Harvard Apparatus, Holliston, MA, USA).

Blood was collected from the trunk into tubes and allowed to clot for 30 min at room temperature. Serum was obtained by centrifuging the samples at 2,000 × g for 15 min at 14 °C and stored at −80 °C for subsequent analysis. The liver, visceral and subcutaneous adipose tissue, and small intestine (jejunum) were carefully excised and weighed. The jejunum was selected as the primary site of nutrient absorption and chylomicron assembly, and is known to exhibit early and robust responses to a high-fat diet (Zhu et al., 2021). Tissue samples for molecular analysis were frozen in liquid nitrogen and stored at −80 °C, while those for histological analysis were fixed in 4% paraformaldehyde.

All procedures were in accordance with the ECC Directive (2010/63/EU) and were approved by the Ethics Committee for the Use of Laboratory Animals of the Ministry of Agriculture, Forestry and Water Management of the Republic of Serbia (reference number 119-01-4/11/2020–09 dated 28 October 2020).

### 2.4 Fecal microbiota profiling and bioinformatics analysis

Fecal samples from each animal were collected in sterile tubes after spontaneous defecation, frozen in liquid nitrogen and stored at −80 °C until further analysis.

Genomic bacterial DNA was extracted from the feces using the Quick-DNA™ Fecal/Soil Microbe Miniprep Kit (Zymo Research, Irvine, CA, USA). The extracted DNA was sent to Novogen Co. (China) for commercial paired-end V3–V4 16S rRNA next-generation sequencing using universal primers 341F and 806R with sequences 5′-CCTAYGGGRBGCASCAG-3′ and 5′-GGACTACNNGGGTATCTAAT-3′. The resulting raw sequencing data were processed using the QIIME2 pipeline (qiime2-amplicon-2024.5) (Bolyen et al., 2019). Primer and barcode sequences were removed using the q2-cutadapt plugin. For quality control, low-quality reads and chimeric sequences were removed, overlapping reads were merged and sequences were denoised. These measures were performed with the q2-dada2 plugin (Martin, 2011; Callahan et al., 2016). The taxonomic assignment of ASVs was determined with the q2-feature-classifier plugin, which applies a Naive Bayes classifier trained on the SILVA 138.2 reference database (Quast et al., 2013; Bokulich et al., 2018). Subsequent analyzes were performed in the R environment (https://www.R-project.org/) after importing the data with the file2meco package. The microbial community was analyzed using the microeco package (Liu et al., 2021; 2022). Alpha diversity was assessed by various indices, including Observed Features, Shannon Diversity Index, Fisher’s Alpha and Pielou’s Evenness Index. Beta diversity was assessed using Bray–Curtis and Jaccard dissimilarity metrics and results were visualized using principal coordinate analysis (PCoA), while ANOSIM was used for statistical analysis.

### 2.5 Quantification of short-chain fatty acids (SCFA)

SCFAs were isolated from fecal samples according to a modified protocol by Zhang et al. (Zhang et al., 2019). In brief, 50 mg of feces was weighed and mixed with 0.5 mL of deionized water. Samples were vortexed for 30 s and then sonicated in an ice-water ultrasonic bath at 40 kHz for 10 min, which was followed by centrifugation at 10,000 × g for 15 min at 4 °C. Subsequently, 400 µL of the supernatant was transferred to a new tube, mixed with 40 µL of 5 M HCl and vortexed. The mixture was extracted with 400 µL anhydrous diethyl ether (DE) (1:1, v/v), vortexed, incubated on ice for 5 min and centrifuged at 10,000 × g for 5 min at 4 °C. The DE layer (upper phase) was carefully transferred to a new tube containing anhydrous sodium sulfate (Na_2_SO_4_) to remove residual water. This extraction process was repeated three times to ensure efficient SCFA recovery. The DE layers were pooled and derivatized for further analysis.

Profiling of eight SCFAs (acetic acid, propionic acid, 2-methyl propionic acid, butyric acid, valeric acid, 4-methylvaleric acid, hexanoic acid and heptanoic acid) in fecal samples was performed using an Agilent 8890 gas chromatography (GC) system coupled to a Mass Selective Detector (5977B GC/MSD, Agilent Technologies, Santa Clara, CA) and connected to an automated sample extraction and enrichment platform (Centri, Markes International Ltd., Bridgend, UK). The derivatization was performed by transferring 100 µL of the DE extract to a GC vial and then adding 1 µL of N,O-bis(trimethylsilyl)trifluoroacetamide (BSTFA) reagent, which was followed by rigorous shaking. Before being injected into the GC/MS instrument, the reaction mixtures were kept in GC vials at room temperature for 15 min to complete the derivatization process. The same derivatization procedure was adopted for the standards. Chromatographic separations of SCFAs were performed for 21 min on an HP-5MS column (30 m × 0.25 mm, 0.25 µm film thickness; Agilent Technologies, Santa Clara, USA), using Helium (99.999%, The Linde Group, Ireland) as the carrier gas at a flow rate of 1.6 mL/min. The transfer line was heated to 280 °C and the detector temperature was set to 270 °C. The column temperature was linearly increased from 40 °C to 300 °C at a rate of 20 mL/min and held isothermally at 240 °C for the following 5 min. Mass spectra were recorded in positive EI mode (+70 eV) with the EI source temperature set to 280 °C, adopting a combined Single Ion Monitoring (SIM)/SCAN mode. Untargeted tracking of compounds in the 45–500 amu range was performed in the SCAN mode. Identification of compounds was performed in Agilent MassHunter Qualitative Analysis 10.0. by comparing their mass spectra and retention times (Rt) with those of the respective standards as well as with spectra from the NIST (vers. 2.4) library. The quantitative analysis of the SCFAs of interest was based on the SIM data aligned to the following masses: 60.06, 74.08, 88.11, 102.13, 116.16, and 130.18 (Supplementary Table S1). Quantification was performed in Agilent MassHunter Quantitative Analysis 10.2. using the external standard method, and adopting the calibration curves of the pure standards, which all showed good linearity with r^2^ values above 0.99 (peak areas vs. concentration). The standards for acetic acid, propionic acid, 2-methyl propionic acid, butyric acid, valeric acid, 4-methylvaleric acid, hexanoic acid and heptanoic acid were purchased commercially from Sigma-Aldrich (St. Louis, MO, United States, Supplementary Table 1).

### 2.6 Intestinal permeability assay

*In vivo* intestinal permeability was determined by measuring the concentration of fluorescein isothiocyanate (FITC)-dextran (FD4-1G, Sigma-Aldrich, St. Louis, USA) in the blood 5 days before sacrifice (Woting and Blaut, 2018). Mice were fasted for 4 hours before and after administration of 150 µL of 80 mg/mL FITC-dextran solution via oral gavage. Blood was collected retro-orbitally 4 hours after gavage and centrifuged at 3,000 × g for 10 min at 4 °C (Eppendorf 5804/R, Hamburg, Germany). The obtained plasma samples were diluted in five volumes (w/v) of PBS and the fluorescence intensity was measured at 530 nm with excitation at 485 nm using the Synergy H1 microplate reader (BioTek instruments, Winooski, USA).

### 2.7 Assessment of systemic glucose tolerance

The concentrations of fasting serum insulin and glucose were used for the calculation of homeostasis model assessment (HOMA) index using following formula: [insulin (mIU/L) × glucose (mmol/L)]/22.5 (Matthews et al., 1985).

Intraperitoneal glucose tolerance test (ipGTT) was performed 3 days before the end of the experiment. The mice were fasted for 4 hours, after which glucose was administered intraperitoneally (1 g/kg). Glucose levels were measured using Accu-Chek^®^ strips (F. Hoffmann-La Roche AG, Basel, Switzerland) from whole blood collected from the tip of the tail at 0, 15, 30, 60, 90, 120 and 150 min after the glucose administration. The area under the concentration vs. time curve (AUC glucose 0–150 min, mmol/L vs. minute) was calculated using the trapezoidal rule (Jaki and Wolfsegger, 2009).

### 2.8 Measurement of biochemical parameters

The concentration of triglycerides, cholesterol, aspartate aminotransferase (AST), alanine aminotransferase (ALT), gamma-glutamyl transferase (GGT), FFA and beta-hydroxybutyrate (BHB) in the serum were measured on a semi-automatic biochemistry analyzer Mindray BS-240 (Mindray, Shenzhen, China) by using commercially available reagents (triglycerides: 11,528, cholesterol: 11,505, AST: 11,531, ALT: 11,533, GGT: 11,510, FFA: 11,840, BHB: 12,525, BioSystems, Barcelona, Spain).

Leptin and insulin concentrations were measured using commercial available ELISA kits (Leptin: #EZML-82K, Insulin: #EZRMI-13K, Millipore Corporation, St. Louis, USA). The absorbance at 450 nm (with a reference at 590 nm) for both parameters was determined using a spectrophotometer (Multiskan Spectrum, Thermo Electron Corporation, Waltham, USA). The 4 PL curve was generated using GraphPad Prism 8 software (GraphPad Software, Inc., USA), which was then used to calculate the concentrations expressed in ng/mL. The sensitivity of the leptin assay was 0.05 ng/mL, with an intra-assay CV of 1.64% and an inter-assay CV of 3.96%. The insulin assay had a sensitivity of 0.1 ng/mL (17.5 pM) with a sample volume of 10 μL, and the intra-assay coefficient of variation was 1.92%.

### 2.9 Histological analysis of the liver and small intestine

Liver and small intestine (jejunum) specimens were fixed in a 4% paraformaldehyde solution for 24 h and processed with an ethanol gradient for dehydration, xylene for clarification and paraffin embedding. The paraffin blocks were cut into 5 µm thick slices and stained with hematoxylin and eosin using standard procedures (Fischer et al., 2008). The histological sections were examined using a Leitz DMRB light microscope equipped with a Leica MC190HD camera and Leica Application Suite (LAS) 4.11.0 software (Leica Microsystems, Wetzlar, Germany) at ×10 magnification. Morphometric analysis of the small intestine (including villus length, crypt depth, mucosal, submucosal and muscularis externa thickness) was performed using ImageJ software (https://imagej.nih.gov/ij/). The analysis was performed in a blinded fashion, with three sections (at 100 µm intervals) per animal.

### 2.10 Preparation of cytoplasmic and nuclear fractions of the liver and total protein fraction of the small intestine

Liver samples were thawed, weighed, and homogenized using a Janke-Kunkel Ultra Turax (30 s/30 s pause/30 s) in four volumes (w/v) of ice-cold homogenization buffer (20 mM Tris-HCl pH 7.2, 10% glycerol, 50 mM NaCl, 1 mM EDTA-Na_2_, 1 mM EGTA-Na_2_, 2 mM DTT, and phosphatase and protease inhibitors). The homogenates were filtered through gauze and centrifuged at 2,000 × g for 15 min at 4 °C (Eppendorf 5804/R, Hamburg, Germany). The supernatants were collected for further processing to isolate cytoplasmic fractions, while the pellets were retained to obtain nuclear fractions. The supernatants were ultracentrifuged at 200,000 × g for 90 min at 4 °C (Beckman L7-55, Brea, USA), with the final supernatants used as the cytoplasmic fractions. To prepare the nuclear fractions, pellets obtained from the first centrifugation were washed twice with HEPES buffer (25 mM HEPES pH 7.6, 1 mM EDTA-Na_2_, 1 mM EGTA-Na_2_, 10% glycerol, 50 mM NaCl, 2 mM DTT, and phosphatase and protease inhibitors) by centrifugation at 4,000 × g for 10 min at 4 °C (Eppendorf 5804/R, Hamburg, Germany). The resulting pellets were resuspended in NUN buffer (25 mM HEPES, pH 7.6, 1 M urea, 300 mM NaCl, 1% Nonidet P-40, and protease and phosphatase inhibitors), and incubated for 90 min on ice with continuous shaking and frequent vortexing. After centrifugation at 8,000 × g for 10 min at 4 °C (Eppendorf 5804/R, Hamburg, Germany), the supernatants were collected as nuclear fractions (Vasiljević et al., 2013). The extraction of cellular fractions was carried out on 4 °C and the samples were stored at −80 °C. Protein concentrations of liver cell fractions were determined using the method described by Spector (Spector, 1978), with bovine serum albumin as the standard.

The total protein fraction from the small intestine was extracted using the TRIzol® protocol (Chomczynski and Sacchi, 1987), following the manufacturer’s instructions. After RNA precipitation, ethanol was added to the remaining organic phase, followed by centrifugation at 2000 *g* for 5 min at 4 °C (Eppendorf 5804/R, Hamburg, Germany). The protein fraction was precipitated from the phenol-ethanol supernatant using acetone and centrifuged at 12,000 × g for 10 min at 4 °C (Eppendorf 5804/R, Hamburg, Germany). The protein pellets were resuspended in 0.3 M guanidine hydrochloride in 95% ethanol with 2.5% glycerol, sonicated on ice, and washed with the same buffer. After pelleting the proteins by centrifugation at 8,000 × g for 5 min at 4 °C (Eppendorf 5804/R, Hamburg, Germany), the pellets were dissolved in lysis buffer containing 2.5 mM Tris-HCl pH 6.8, 2% SDS, 10% glycerol, and 50 mM DTT. The isolates were stored at −80 °C until further analysis. The protein concentrations of total protein fractions of the intestine were measured by Pierce BCA Protein Assay Kit (A65453, ThermoFisher Scientific, Waltham, USA) according to manufacturer’s instructions.

### 2.11 Western blot analysis

Cellular extracts were boiled in Laemmli buffer for 5 min (Laemmli, 1970). For gel electrophoresis, 40 μg of protein from whole cell extracts and cytoplasmatic fractions, and 60 μg of protein from the nuclear fractions were loaded onto 8% sodium dodecyl sulfate-polyacrylamide gels, which were run using the Mini-Protean II Electrophoresis Cell System (Bio-Rad Laboratories, Hercules, USA). Following electrophoresis, proteins were transferred from the gels to polyvinylidene fluoride (PVDF) membranes (Immobilon-FL, Merck Millipore, Billerica, USA) using the Mini Trans-Blot cell system (Bio-Rad Laboratories, Hercules, USA). The membranes were then blocked with 2% BSA in PBS buffer, washed, and incubated overnight at 4 °C with the following primary antibodies: anti-SREBP-1c (1:500, sc-366, Santa Cruz Biotechnology), anti-perilipin 2 (1:1000, NB110-40877, Novus Biologicals), anti-NOD-like receptor protein 3 (NLRP3) (1:500, NBP2-12446, Novus Biologicals), anti-glucocorticoid receptor (GR) (1:250, sc-8992, Santa Cruz Biotechnology), anti-zonula occludens-1 (ZO-1) (1:1000, 40-2200, Thermo Fisher Scientific), anti-occludin (1:500, ab216327, Abcam). The membranes were then washed and incubated with the appropriate horseradish peroxidase-conjugated secondary antibodies: Anti-Rabbit IgG (1:20,000, NB7160, Novus Biologicals) and Anti-Mouse IgG (1:30,000, ab97046, Abcam). Anti-β-actin (1:10,000, ab8227, Abcam) was used as the loading control for the cytoplasmic fraction of the liver and total protein fraction of the intestine, while anti-Lamin B1 (1:1000, sc-374015, Santa Cruz) were used for the nuclear fraction. Immunoreactive protein bands were visualized using the chemiluminescence method with the iBright CL1500 Imaging System (Supplementary Data Sheet 1), and quantitative analysis was performed using iBright Analysis Software (Thermo Fisher Scientific, Waltham, USA).

### 2.12 Quantitative polymerase chain reaction (qPCR)

Total RNA was extracted from the intestine using TRIzol® reagent (Ambion, Life Technologies, Austin, USA), according to manufacturer’s protocol. The RNA concentration was measured by assessing the optical density at 260 nm using the NanoPhotometer N60 (Implen, Munich, Germany). Complementary DNA (cDNA) was synthesized using the Applied Biosystems High-Capacity cDNA Reverse Transcription Kit (Thermo Fisher Scientific, Waltham, USA) the cDNA samples were stored at −80 °C until further analysis.

Gene expression analysis was carried out using the Power SYBR® Green PCR Master Mix (Applied Biosystems, Thermo Fisher Scientific, Waltham, USA). Specific primers (Microsynth, Balgach, Switzerland) were used for selective amplification of the examined genes (Table 1). Quantitative normalization of cDNA was performed using hypoxanthine-guanine phosphoribosyl transferase (*Hprt*) as an endogenous control. All qPCR reactions were performed in duplicate in 10 µL volumes with 20 ng of cDNA template using the Quant Studio™ 3 (Applied Biosystems, Thermo Fisher Scientific, Waltham, USA). The thermal cycling conditions were as follows: 2 min at 50 °C, 10 min at 95 °C, followed by 45 cycles of 95 °C for 15 s and 60 °C for 60 s. Relative gene expression was quantified using the comparative 2^−ΔΔCt^ method (Livak and Schmittgen, 2001). Data analysis was performed with Quant Studio™ Design and Analysis software v1.4.0 (Applied Biosystems, Thermo Fisher Scientific, Waltham, USA) with a confidence level of 95% (p ≤ 0.05).

**TABLE 1:** Primer sequences used for quantitative real-time PCR analysis.

Gene	Forward primer (5′→3′)	Reverse primer (5′→3′)
*Cd36*	CAT TTG CAG GTC TAT CTA CG	CAA TGT CTA GCA CAC CAT AAG
*CerS6*	GAG ATT AGA AGG GCT CTC CA	CAC ATG CTC TCA CAG AAC CT
*DegS1*	ATG TCT TTG GCA GCT GCC TT	CAT TCC AAA CCA GCG GTT CC
*Fas*	TTG CTG GCA CTA CAG AAT GC	AAC AGC CTC AGA GCG ACA AT
*Scd1*	CTG TAC GGG ATC ATA CTG GTT C	GCC GTG CCT TGT AAG TTC TG
*Dgat1*	GTG CAC AAG TGG TGC ATC AG	CAG TGG GAC CTG AGC CAT CA
*Mttp*	TGA GCG GCT ATA CAA GCT CAC	CTG GAA GAT GCT CTT CTC GC
*Tlr4*	ATC ATC CAG GAA GGC TTC CA	GCT AAG AAG GCG ATA CAA TTC
*Myd88*	TCA TGT TCT CCA TAC CCT TGG T	AAA CTG CGA GTG GGG TCA G
*Tnfα*	CTC AGC CTC TTC TCA TTC CTG CT	CTG ATG AGA GGG AGG CCA TT
*Il-1β*	CAG GCT CCG AGA TGA ACA AC	AGG CCA CAG GTA TTT TGT CG
*Hprt1*	TCC TCC TCA GAC CGC TTT T	CCT GGT TCA TCA TCG CTA ATC

Abbreviations: *Cd36* – fatty acid translocase CD36, *CerS6 -* ceramide synthase 6*, DegS1 -* dihydroceramide desaturase 1, *Fas* - fatty acid synthase, *Scd1* - stearoyl-CoA, desaturase 1, *Dgat1* - diacylglycerol O-acyltransferase 1, *Mttp* - microsomal triglyceride transfer protein, *Tlr4* - toll like receptor 4, *Myd88* - myeloid differentiation primary response 88, *Tnfα* - tumor necrosis factor alpha, *Il-1β*–interleukin 1β*, Hprt1* - hypoxanthine-guanine phosphoribosyl transferase 1.

### 2.13 Statistical analysis

The normality of the data was evaluated using the Shapiro–Wilk test. Data that followed a normal distribution were analyzed using One-way ANOVA followed by the Tukey *post hoc* test. Data that deviated from a normal distribution were analyzed with the Kruskal–Wallis *H* test, followed by Dunn’s *post hoc* test. A p-value of less than 0.05 was considered statistically significant. All statistical analyses were conducted using GraphPad Prism 8 (GraphPad Software, Inc., USA). Beta diversity analysis was performed using ANOSIM. Data visualization was performed using the R programming language (version 4.1.0) and GraphPad Prism 8.

## 3 Results

### 3.1 LHBGRA43 decreased body, liver and subcutaneous adipose tissue mass of mice fed a high-fat diet

The animals in both groups fed a high-fat diet had increased caloric intake regardless of the LHBGRA43 treatment (p < 0.001, Table 2). Although body mass increased in both groups on the high-fat diet (p < 0.001, HFD vs. C; p < 0.01, HFD + B vs. C), the HFD + B group had a lower body mass compared to the HFD group (p < 0.01). Decreased liver mass was also detected after LHBGRA43 treatment (p < 0.01, HFD + B vs. HFD, Table 2). Although liver mass was increased in the HFD group compared to the control (p < 0.05), the liver-to-body ratio in this group remained unchanged, while it was decreased in the HFD + B group compared to the HFD (p < 0.05) and control group (p < 0.01). On the other hand, probiotic treatment had no effect on visceral adipose tissue mass, which was increased in both high-fat diet groups (p < 0.001). In accordance with this result, increased serum leptin concentration (p < 0.001, Table 3) was observed in the animals on the high-fat diet, regardless of treatment with LHBGRA43. However, the mass of subcutaneous adipose tissue was also increased in both high-fat diet groups (p < 0.001, HFD vs. C; p < 0.01, HFD + B vs. C), but it was significantly lower after LHBGRA43 treatment compared to the HFD (p < 0.05, Table 2).

**TABLE 2:** The effect of LHBGRA43 on morphological parameters.

Parameter	C	HFD	HFD + B
Caloric intake (kcal/day/animal)	9.32 ± 0.40	14.50 ± 0.32***	14.64 ± 0.42***
Body mass (g)	34.25 ± 1.05	46.88 ± 1.53***	40.50 ± 0.80**^##^
Liver mass (g)	1.19 ± 0.02	1.53 ± 0.11*	1.14 ± 0.06^##^
Liver-to-body mass ratio (x1000)	33.83 ± 1.26	32.13 ± 1.40	27.48 ± 0.92**^#^
VAT mass (g)	1.97 ± 0.24	4.45 ± 0.20***	4.15 ± 0.21***
SAT mass (g)	1.29 ± 0.13	3.64 ± 0.36***	2.68 ± 0.24**^#^

The data are presented as mean ± SEM (n = 8 animals per group). Asterisk (*) indicates significant differences in comparison to the C group (*p < 0.05, **p < 0.01, ***p < 0.001), while hash sign (#) indicates significant differences between HFD and HFD + B (^#^p < 0.05, ^##^p < 0.01). Abbreviations: C, control; HFD, high-fat diet; HFD + B, combination of high-fat diet and oral supplementation with the bacterial strain LHBGRA43; VAT, visceral adipose tissue; SAT, subcutaneous adipose tissue.

**TABLE 3:** The effect of LHBGRA43 on biochemical parameters.

Parameter	C	HFD	HFD + B
ALT (U/L)	48.56 ± 2.93	95.96 ± 18.70*	67.35 ± 11.00
AST (U/L)	380.28 ± 48.71	290.30 ± 25.07	272.36 ± 12.89
GGT (U/L)	2.80 ± 0.41	2.31 ± 0.22	2.29 ± 0.34
TG (mmol/L)	1.98 ± 0.09	1.98 ± 0.15	1.82 ± 0.15
FFA (mmol/L)	1.70 ± 0.11	1.38 ± 0.05	1.38 ± 0.12
Cholesterol (mmol/L)	4.10 ± 0.39	5.60 ± 0.30*	5.50 ± 0.33*
Glucose (mmol/L)	9.46 ± 0.67	12.06 ± 0.42**	11.83 ± 0.48*
Insulin (ng/mL)	2.41 ± 0.36	7.25 ± 1.62**	6.30 ± 0.72*
AUC-IPGTT	1510.00 ± 88.00	2085.75 ± 123.92**	1901.00 ± 87.11*
HOMA index	0.31 ± 0.05	1.17 ± 0.26**	1.06 ± 0.14*
Leptin (ng/mL)	10.43 ± 1.91	38.42 ± 4.72***	35.90 ± 3.83***
BHB (mmol/L)	0.14 ± 0.02	0.21 ± 0.02	0.18 ± 0.06

Data are presented as mean ± SEM (n = 8 animals per group). Asterisk (*) indicates significant differences compared to the C group (*p < 0.05, **p < 0.01, ***p < 0.001). Abbreviations: C, control; HFD, high-fat diet; HFD + B, combination of high-fat diet and oral supplementation with the bacterial strain LHBGRA43; ALT, alanine aminotransferase; AST, aspartate aminotransferase; GGT, gamma-glutamyl transferase; TG, triglycerides; FFA, free fatty acids; AUC-IPGTT, area under the curve level obtained from intraperitoneal glucose tolerance test; HOMA, index–homeostasis model assessment index; BHB, beta-hydroxybutyrate.

### 3.2 LHBGRA43 reversed increased ALT level in mice fed a high-fat diet

Serum ALT concentration, the main marker of liver damage, was increased on the high-fat diet (p < 0.05, Table 3) and improved after treatment with the probiotic, which returned ALT level to the level not significantly different than in the control group. Neither the high-fat diet nor the supplemented bacteria had effects on AST, GGT, triglyceride and FFA concentrations, while cholesterol levels were elevated in both high-fat diet groups (p < 0.05, Table 3), with no effect of LHBGRA43. In the animals fed with a high-fat diet without LHBGRA43, glucose and insulin levels, as well as the indicators of systemic glucose intolerance, the HOMA index and the AUC-IPGTT value, were elevated compared to the control animals (p < 0.01, Table 3). Although these parameters were also increased in the HFD + B group compared to the control animals this increase was slightly lower (p < 0.05, Table 3). The BHB concentration, indicator for energy production from fatty acids in the β-oxidation process, was not altered in the experimental groups.

### 3.3 LHBGRA43 suppressed lipogenesis and decreased fatty acid influx and ceramide synthesis in the liver

**FIGURE 1:**
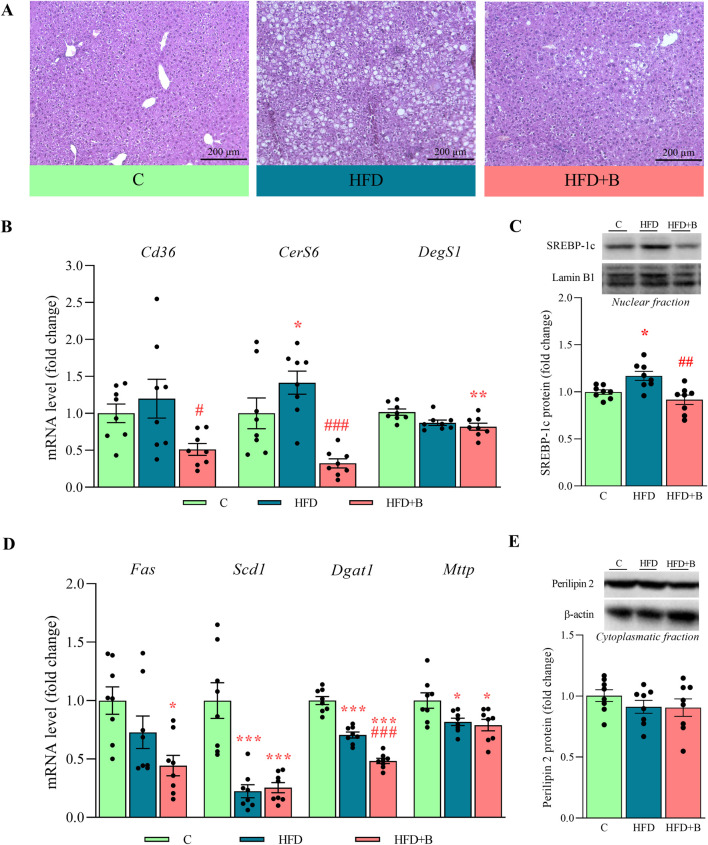
Histological analysis of the liver and the level of markers of hepatic lipid metabolism. Representative micrographs of hematoxylin-eosin-stained liver sections **(A)**, mRNA levels of *Cd36*, *CerS6* and *DegS1* **(B)**, representative Western blots and protein level of SREBP-1c **(C)**, mRNA levels of *Fas*, *Scd1*, *Dgat1* and *Mttp* **(D)**, representative Western blots and protein level of perilipin 2 **(E)**. Histological sections were examined at ×10 magnification. Scale bars are 200 μm. Quantification of mRNA levels in the liver was done relative to the amount of *Hprt1*. For Western blot analyses, lamin-B1 and β-actin were used as loading controls for the nuclear and cytoplasmatic fractions, respectively. Data are presented as mean ± SEM (n = 8 animals per group). One-way ANOVA followed by a Tukey *post hoc* test or a non-parametric Kruskal–Wallis *H* test followed by a Dunn’s *post hoc* test was used to assess statistical significance. Asterisk (*) indicates significant differences compared to the C group (*p < 0.05, **p < 0.01, ***p < 0.001), while hash sign (#) indicates significant differences between HFD and HFD + B (^#^p < 0.05, ^##^p < 0.01, ^###^p < 0.001). Abbreviations: C – control, HFD–high-fat diet, HFD + B–combination of high-fat diet and oral supplementation with the bacterial strain LHBGRA43, SREBP-1c - sterol regulatory element-binding protein 1, *Cd36* – fatty acid translocase CD36, *CerS6 -* ceramide synthase 6*, DegS1 -* dihydroceramide desaturase 1, *Fas* - fatty acid synthase, *Scd1* - stearoyl-CoA desaturase 1, *Dgat1* - diacylglycerol O-acyltransferase 1, *Mttp* - microsomal triglyceride transfer protein, *Hprt1* - hypoxanthine-guanine phosphoribosyl transferase 1.

Liver sections stained with hematoxylin and eosin showed that treatment with a high-fat diet led to the development of steatosis without concomitant fibrosis. Oral administration of LHBGRA43 reduced the accumulated lipid droplets and cleared steatosis despite the high-fat diet (Figure 1A). Examination of lipid metabolism in the liver showed that treatment with the probiotic led to a reduced mRNA level of the fatty acid transporter *Cd36*, which indicates a reduced influx of fatty acids from the bloodstream into the liver in the HFD + B group compared to the HFD group (p < 0.05, Figure 1B). Ceramide synthase 6 (*CerS6*) mRNA level was increased in the liver of the animals fed high-fat diet (p < 0.05) and significantly decreased after treatment with LHBGRA43 compared to the HFD group (p < 0.001, Figure 1B). Probiotic administration also decreased *DegS1* mRNA levels compared to the control animals (p < 0.01, Figure 1B), indicating that the synthesis of ceramides was attenuated.

The protein level of sterol regulatory element-binding protein 1c (SREBP-1c), a key regulator of *de novo* lipogenesis, was increased in the nuclear fraction of the liver in animals fed a high-fat diet compared to controls (p < 0.05). Treatment with LHBGRA43 markedly reduced nuclear SREBP-1c levels compared to the HFD group (p < 0.01, Figure 1C). The probiotic treatment also significantly decreased *Fas* mRNA level in compare to control (p < 0.05, Figure 1D). Suppressed *de novo* lipogenesis was accompanied with decreased *Scd1* (p < 0.001) and *Mttp* (p < 0.05) mRNA levels in both high-fat diet groups compared to controls, regardless of the LHBGRA43 treatment (Figure 1D). The expression of *Dgat1* was also decreased in the HFD group compared to controls (p < 0.001), but the bacteria reduced its mRNA level in the liver even further (p < 0.001, for both HFD + B vs. C and HFD + B vs. HFD, Figure 1D). Although the synthesis of triglycerides was decreased, the protein level of perilipin 2 remained unchanged (Figure 1E).

### 3.4 LHBGRA43 decreased the expression of proinflammatory mediators in the liver

**FIGURE 2:**
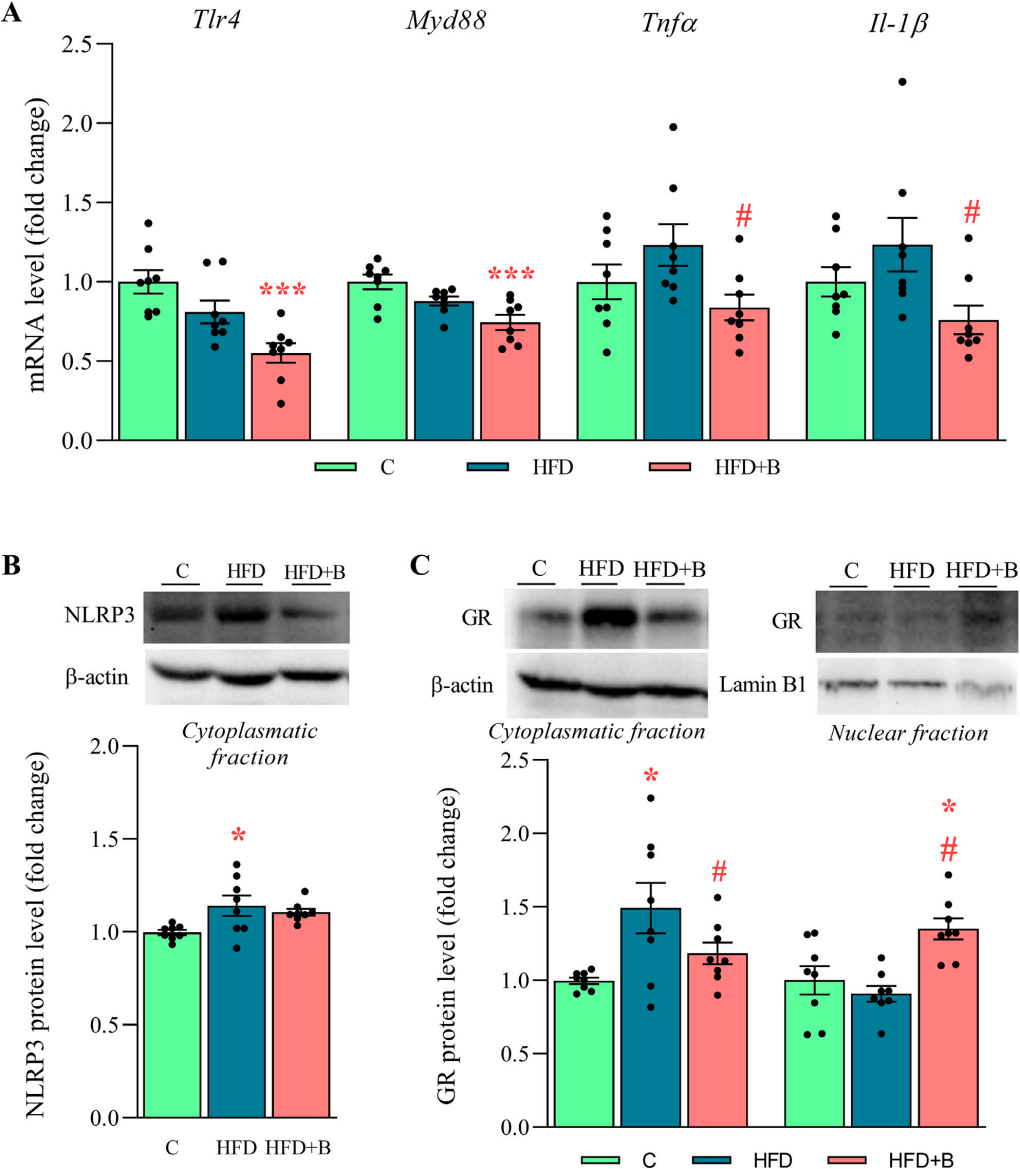
The level of proinflammatory mediators, NLRP3 inflammasome and subcellular distribution of GR in the liver. The mRNA levels of *Tlr4*, *Myd88*, *Tnfα* and *Il-1β* **(A)**, representative Western blots and protein levels of NLRP3 **(B)** and GR **(C)**. Quantification of mRNA levels in the liver was done relative to the amount of *Hprt1*. β-actin and lamin-B1 were used as loading controls for the cytoplasmatic and nuclear fractions, respectively. Data are presented as mean ± SEM (n = 8 animals per group). One-way ANOVA followed by a Tukey *post hoc* test or a non-parametric Kruskal–Wallis *H* test followed by a Dunn’s *post hoc* test was used to assess statistical significance. Asterisk (*) indicates significant differences in regard to the C group (*p < 0.05, ***p < 0.001), while hash sign (#) indicates significant differences between HFD and HFD + B (^#^p < 0.05). Abbreviations: C – control, HFD–high-fat diet, HFD + B–combination of high-fat diet and oral supplementation with the bacterial strain LHBGRA43, *Tlr4* - toll like receptor 4, *Myd88* - myeloid differentiation primary response 88, *Tnfα* - tumor necrosis factor alpha, *Il-1β*–interleukin 1β, NLRP3 - NOD-like receptor protein 3, GR–glucocorticoid receptor, *Hprt1* - hypoxanthine-guanine phosphoribosyl transferase 1.

The expression of Toll-like receptor 4 (*Tlr4*) and its effector Myeloid differentiation primary response 88 (*Myd88*) was evaluated, as their function may depend on the composition of the gut microbiota and the concentration of FFA. Treatment with LHBGRA43 decreased both *Tlr4* and *Myd88* expression compared to the control animals (p < 0.001 for both, Figure 2A). The protein level of the NLRP3 inflammasome was increased in the HFD group compared to the C group (p < 0.05, Figure 2B), while slightly increased expression of the proinflammatory cytokines *Tnfα* and *Il-1β* did not reach statistical significance in this group compared to control. However, after the treatment with LHBGRA43, proinflammatory cytokines *Tnfα* and *Il-1β* were significantly decreased compared to the HFD group (p < 0.05 for both *Tnfα* and *Il-1β*, Figure 2A), confirming the anti-inflammatory properties of LHBGRA43. Interestingly, subcellular translocation of the glucocorticoid receptor, which has strong anti-inflammatory properties, was also stimulated in the HFD + B group, as its level decreased in the cytoplasmatic fraction compared to the HFD group and increased in the nuclear fraction compared to both the C and HFD groups (p < 0.05, Figure 2C).

### 3.5 LHBGRA43 administration improved integrity of small intestine barrier

**FIGURE 3:**
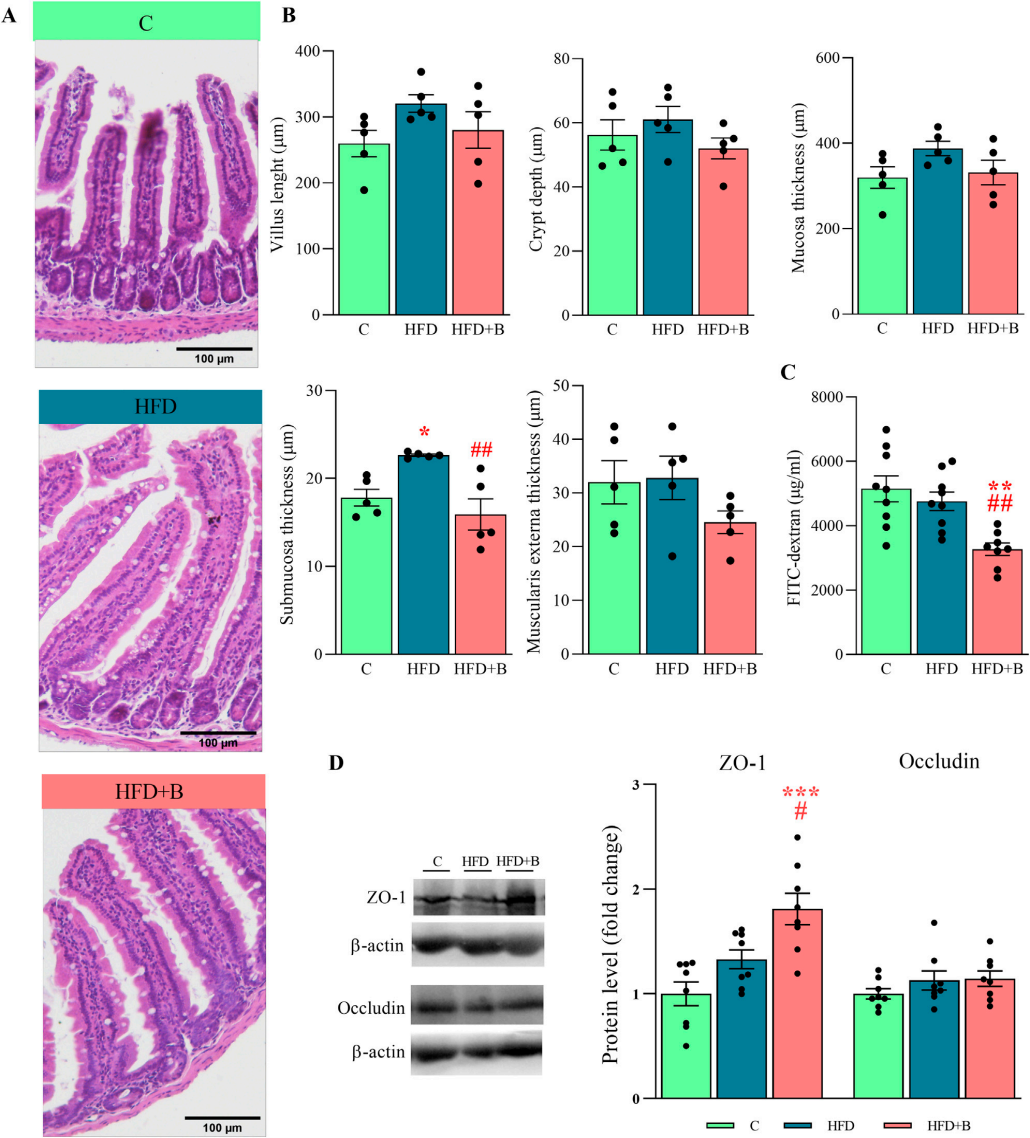
Histological analysis, integrity and permeability of the small intestine and the level of tight junction proteins. Representative micrographs of hematoxylin-eosin-stained small intestine sections **(A)**, villus length, crypt depth, mucosa length, submucosa length and muscularis externa length **(B)**, the level of FITC-dextran in the serum **(C)** and representative Western blots and protein levels of ZO-1 and occludin in the total protein fraction of the small intestine **(D)**. Histological sections were examined at ×10 magnification. Scale bars are 100 μm. β-actin was used as loading control for total protein fraction. Data are presented as mean ± SEM (n = 5 animals per group for histological analysis and n = 8 animals per group for the Western blot). One-way ANOVA followed by a Tukey *post hoc* test or a non-parametric Kruskal–Wallis *H* test followed by a Dunn’s *post hoc* test was used to assess statistical significance. Asterisk (*) indicates significant differences in respect to the C group (*p < 0.05, **p < 0.01, ***p < 0.001), while hash sign (#) indicates significant differences between HFD and HFD + B (^#^p < 0.05, ^##^p < 0.01). Abbreviations: C – control, HFD–high-fat diet, HFD + B–combination of high-fat diet and oral supplementation with the bacterial strain LHBGRA43, FITC-dextran - fluorescein isothiocyanate-dextran, ZO-1 – zonula occludens 1.

As shown in the representative micrographs (Figure 3A), the morphology of the small intestine was altered and hypertrophied in both experimental groups that consumed a high-fat diet. However, these changes were less noticeable after supplementation with LHBGRA43. Although the length of the villi, the depth of the crypt, the thickness of the mucosa and the thickness of the muscularis externa tended to decrease in the HFD + B group, these changes were not statistically significant. Only the thickness of the submucosa was significantly increased in the HFD group compared to the controls (p < 0.05) and decreased after administration of the probiotic compared to the group receiving only a high-fat diet (p < 0.01, Figure 3B).

Measurement of FITC-dextran fluorescence in blood serum showed that gut permeability was decreased after treatment with LHBGRA43 compared to both the control and HFD group (p < 0.01, Figure 3C). This result was consistent with the increase in ZO-1 protein in the HFD + B group compared to the control group (p < 0.001) and HFD group (p < 0.05), indicating an improvement in the integrity of the intestinal barrier (Figure 3D).

### 3.6 LHBGRA43 treatment modulated gut microbiota alterations induced by high-fat diet

**FIGURE 4:**
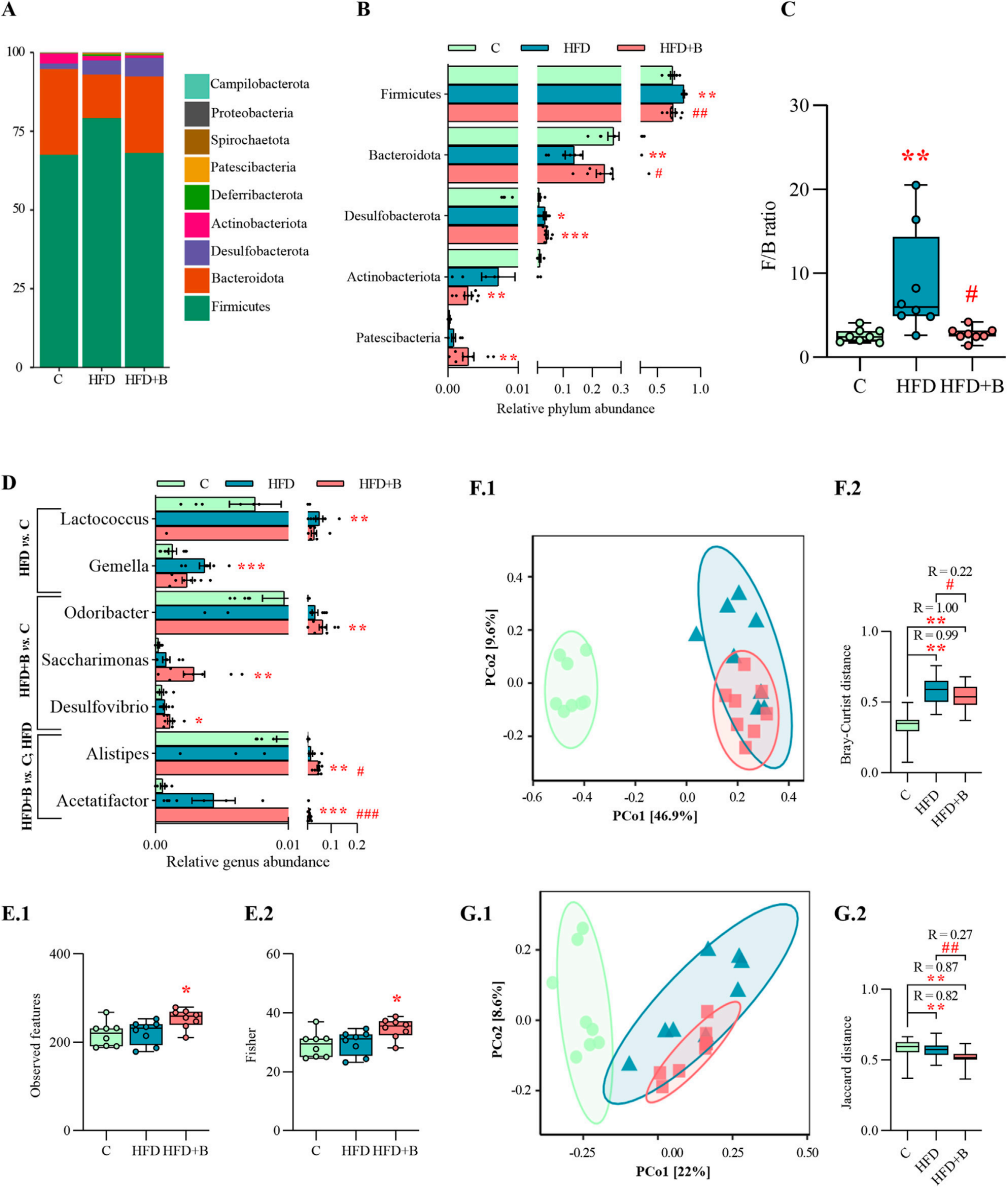
Gut microbiota composition and alpha and beta diversity. Bar chart of relative abundance of bacterial phyla in each group **(A)**, relative abundance of bacterial phyla with statistical analyses **(B)**, *Firmicutes/Bacteroidota* (F/B) phyla ratio **(C)**, relative abundance of bacterial genera with statistical analyses **(D)**, Observed species index **(E1)**, Fisher index **(E2)**, PCoA based on Bray-Curtis distance **(F1)** and ANOSIM analysis **(F2)**, PCoA based on Jaccard distance **(G1)** and ANOSIM analysis **(G2)**. Data are presented as mean ± SEM at panels A, B, D, while at panels C, F and E are presented as box-and-whisker plots, with the median shown as a horizontal line and whisker, extending to the minimal and maximal values in each group (n = 8 animals per group). One-way ANOVA followed by a Tukey *post hoc* test or a non-parametric Kruskal–Wallis *H* test followed by a Dunn’s *post hoc* test was used to assess statistical significance. Asterisk (*) indicates significant differences in comparison to the C group (*p < 0.05, **p < 0.01, ***p < 0.001), while hash sign (#) indicates significant differences between HFD and HFD + B (^#^p < 0.05, ^##^p < 0.01, ^###^p < 0.001). Abbreviations: C – control, HFD–high-fat diet, HFD + B–combination of high-fat diet and oral supplementation with the bacterial strain LHBGRA43.

Taxonomic profiling at the phylum level showed a strong effect of the high-fat diet on the composition of the gut microbiota, but also a protective effect of the tested probiotic bacteria (Figure 4A). Feeding with high-fat diet resulted in a significant increase in the relative abundance of *Firmicutes* and a decrease in *Bacteroidota* compared to controls (p < 0.01, Figure 4B), leading to a significantly higher *Firmicutes/Bacteroidota* (F/B) ratio (p < 0.01, Figure 4C). On the other hand, treatment with LHBGRA43 significantly reduced the F/B ratio compared to the HFD group (p < 0.05, Figure 4C). The differences in the relative abundance of genera were observed in both high-fat diet groups (Figure 4D). The relative abundance of *Lactococcus* (p < 0.01) and *Gemella* (p < 0.001) was only increased in the HFD group compared to the C group, while *Odoribacter* (p < 0.01), *Saccharimonas* (p < 0.01) and *Desulfovibrio* (p < 0.05) were only increased in the HFD + B group compared to the C group. The relative frequencies of *Alistipes* (p < 0.01, HFD + B vs. C; p < 0.05 HFD + B vs. HFD) and *Acetatifactor* (p < 0.001, HFD + B vs. C and HFD, Figure 4D) were increased in the LHBGRA43-treated group.

Analysis of alpha diversity showed that the LHBGRA43 treatment increased the number of Observed features and Fisher diversity index compared to the control group (p < 0.05, Figure 4E1, E2). Beta diversity analyses, using Bray–Curtis and Jaccard distance metrics, showed a clear separation of microbial communities between the experimental groups (Figure 4F1, G1). Significant differences were observed in Bray-Curtis distances between HFD and control groups (p < 0.01, R = 0.99) and between HFD + B and the control (p < 0.01, R = 1, Figure 4F2). In addition, treatment with LHBGRA43 resulted in a significant change in microbial composition compared to the HFD group (p < 0.05, R = 0.22). Consistent with these results, Jaccard-based comparisons were significantly different in the HFD group (p < 0.01, R = 0.82) and the HFD + B group compared to the control group (p < 0.01, R = 0.87), and in the HFD + B group compared to the HFD group (p < 0.01, R = 0.27, Figure 4G2).

### 3.7 LHBGRA43 treatment and high-fat diet changed SCFAs fecal profile

**FIGURE 5:**
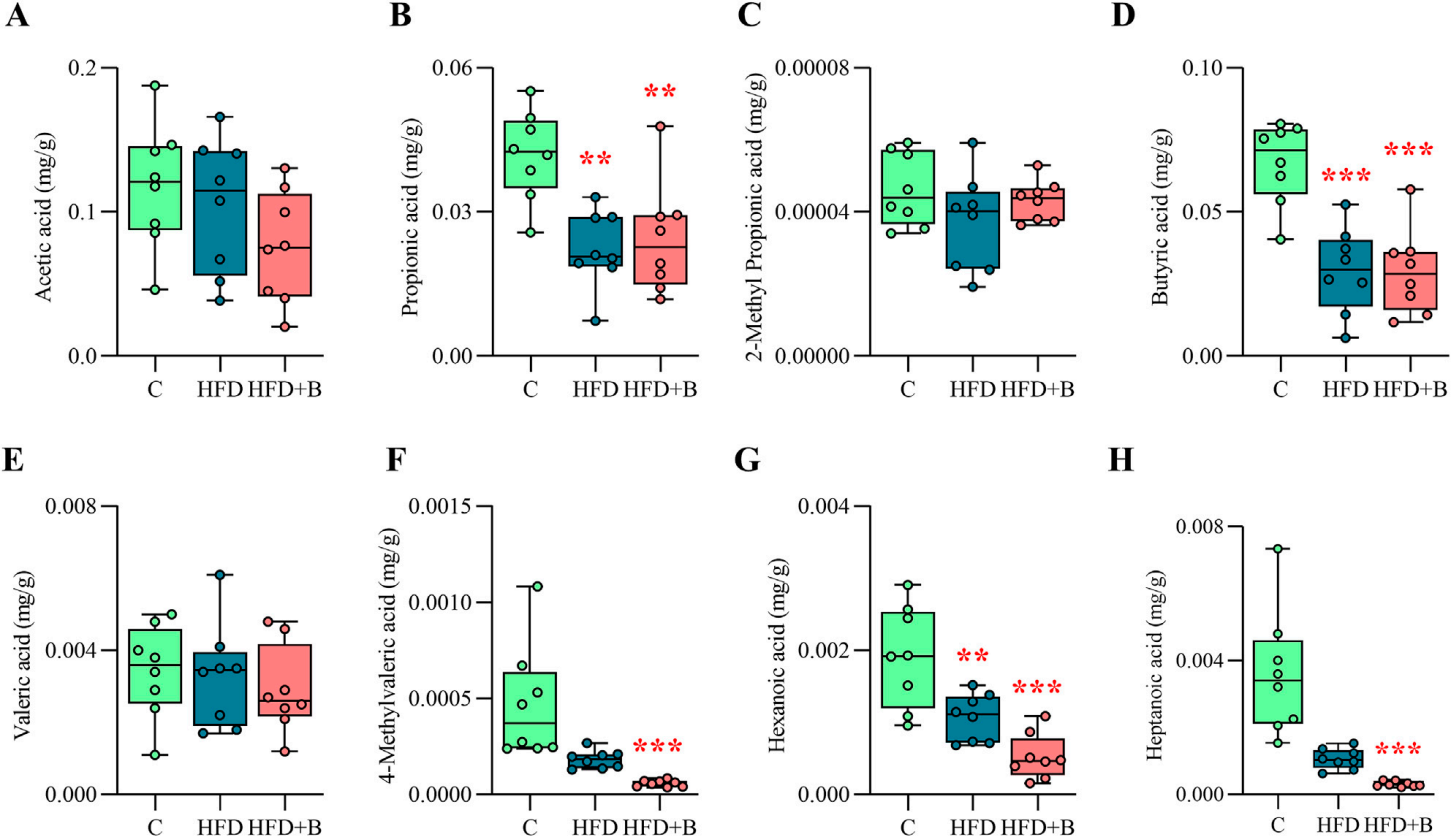
Short-chain fatty acids (SCFA) profile. The concentration of acetic acid **(A)**, propionic acid **(B)**, 2-methyl propionic acid **(C)**, butyric acid **(D)**, valeric acid **(E)**, 4-methylvaleric acid **(F)**, hexanoic acid **(G)** and heptanoic acid **(H)** in fecal samples. Data are presented as box-and-whisker plots, with the median shown as a horizontal line and whisker, extending to the minimal and maximal values in each group (n = 8 animals per group). One-way ANOVA followed by a Tukey *post hoc* test or a non-parametric Kruskal–Wallis *H* test followed by a Dunn’s *post hoc* test was used to assess statistical significance. Asterisk (*) indicates significant differences compared to the C group (**p < 0.01, ***p < 0.001). Abbreviations: C – control, HFD–high-fat diet, HFD + B–combination of high-fat diet and oral supplementation with the bacterial strain LHBGRA43.

As shown in Figure 5, HFD significantly reduced fecal concentrations of propionic acid (p < 0.01, Figure 5B), butyric acid (p < 0.001, Figure 5D) and hexanoic acid regardless of LHBGRA43 administration (p < 0.01, HFD vs. C; p < 0.001, HFD + B vs. C, Figure 5G). However, treatment with LHBGRA43 significantly decreased the concentration of 4-methylvaleric acid (p < 0.001, Figure 5F) and heptanoic acid (p < 0.001, Figure 5H) in comparison to the control animals.

## 4 Discussion

The main finding of this study is that oral administration of the probiotic bacterium LHBGRA43 significantly reduced high-fat diet induced hepatic steatosis. This was associated with suppressed lipogenesis, decreased expression of the fatty acid transporter *Cd36*, reduced expression of enzymes involved in ceramide synthesis and lower levels of proinflammatory mediators in the liver. The ameliorative effect of LHBGRA43 on hepatic lipid accumulation and lipotoxicity may be related to improved intestinal barrier integrity, as evidenced by increased ZO-1 protein levels in the small intestine. The observed reduction in intestinal permeability was associated with a restored *Firmicutes/Bacteroidota* phyla ratio and increased abundance of the genera *Alistipes*, *Acetatifactor* and *Odoribacter* induced by supplementation with LHBGRA43. These findings, together with decreased concentrations of branched-chain 4-methylvaleric acids, support the probiotic potential of the tested bacterium.

To date, the most promising strategy for alleviating MASLD symptoms is to promote weight loss. Several probiotic *Lactobacillus* strains are recognized as regulators of body mass by modulating the gut microbiota, regulating appetite-related hormones and inflammation, and improving lipid metabolism (Wang M. et al., 2020; Ghosh S. et al., 2024; 2025; Oudat and Okour, 2025). Although LHBGRA43 reduced body mass, visceral fat accumulation and leptin secretion were not affected, suggesting that the weight loss mechanisms are not based on appetite regulation or energy production efficiency from food. The observed reduction in body mass appears to result from a decrease in subcutaneous adipose tissue mass and the restoration of liver mass to control levels. The reduction in liver mass after probiotic administration could be related to improved liver metabolism, as indicated by the normal ALT level in the blood of LHBGRA43-treated animals. It has already been shown that probiotic strains of the genera *Lactobacillus* and *Bifidobacterium* can effectively reduce ALT levels, an important marker of ectopic fat accumulation and inflammation in the liver (Lu et al., 2024). However, administration of LHBGRA43 had no effect on blood triglyceride or cholesterol levels, confirming that the effect of probiotics on the lipid profile is less consistent and may be influenced by strain specificity, dosage, and duration of supplementation (Gadelha and Bezerra, 2019). Since LHBGRA43 did not affect glucose tolerance, it can be assumed that this specific strain has a direct effect on hepatic lipid accumulation rather than an indirect effect on the alleviation of steatosis-associated systemic disorders.

The high-fat diet used in this study led to the development of steatosis, the first stage of MASLD, characterized by the accumulation of lipid droplets in the liver with mild metabolic disturbances. This reversible liver condition was successfully reversed by oral supplementation with LHBGRA43, as confirmed by histological analysis. At the molecular level, the elimination of lipids from the liver could be attributed to reduced expression of the hepatic *Cd36* transporter, as well as reductions in SREBP-1c and *Fas* levels. These changes may indicate an attenuation of fatty acid uptake and *de novo* lipogenesis in the liver. This is consistent with growing evidence that *Lactobacillus* strains can ameliorate diet-induced hepatic steatosis by modulating key lipid metabolic pathways (Santos et al., 2024). For example, *Lactobacillus plantarum* LG42 reduced hepatic triglycerides and SREBP-1c levels in obese mice (Park et al., 2014), while *Lactobacillus rhamnosus* GG ameliorated NAFLD by downregulating SREBP-1c protein expression and FAS activity, and reducing host intestinal fatty acid absorption (Arellano-García et al., 2025). Furthermore, administration of LHBGRA43 significantly reduced the expression of *Dgat1* compared to the high-fat diet group, suggesting that the tested strain may directly influence triglyceride formation. However, *Dgat1* expression was also decreased in the high-fat diet group, which together with the reduced *Mttp* expression, showed that steatosis in our animal model was characterized neither by triglyceride production nor by their excretion from the liver. Decreased triglyceride synthesis could be associated with the unchanged perilipin 2 protein level observed in the HFD group, indicating the absence of neutral lipid droplet formation (Doncheva et al., 2023). Instead of neutral lipids, in the high-fat diet group, the hepatic lipid droplets most likely consisted of reactive lipid intermediates, as ceramide synthesis was stimulated. Decreased expression of *CerS6* and *DegS1* observed after treatment with LHBGRA43 led to impaired ceramide synthesis, suggesting that the tested probiotic strain could reduce lipid droplet formation and lipotoxicity associated with excessive ceramide accumulation. *CerS6*-deficient mice are known to be protected from obesity induced by a high-fat diet and exhibit altered hepatic lipid utilization (Turpin et al., 2014). Deletion of the *CerS6* gene has been shown to modulate CD36 function by blocking its trafficking to the plasma membrane and reducing fatty acid influx (Luan and Griffiths, 2006). This mechanism is consistent with the above-mentioned downregulation of *Cd36* and SREBP-1c, suggesting a broader suppression of fatty acid uptake and lipogenesis. Taken together, the results on lipid metabolism support the concept that LHBGRA43 initiates a regulatory feedback loop that limits substrate availability for lipid droplet formation and reduces lipotoxic lipid deposition in the liver, underpinning its therapeutic potential in the prevention or treatment of MASLD.

Although a high-fat diet is often reported to induce inflammatory processes in the liver, the level of proinflammatory markers was not changed in the present study. Similarly, Sutter et al. (2016) did not find significant differences in hepatic *Tlr4* mRNA levels between high-fat and control diet. The absence of increased *Tlr4* gene expression in the HFD group may be attributed to unchanged intestinal permeability, which facilitates the translocation of endotoxins into the circulation, and to normal levels of free fatty acids in the serum. Consequently, downstream inflammatory pathways were not stimulated, confirming that the duration of the applied dietary regimen led to a moderate and reversible stage of metabolically functional steatosis. However, as a result of the positive effect of LHBGRA43 on reducing liver fat, the expression of the pro-inflammatory cytokines *Tnfα* and *Il-1β* was reduced and the level of NLRP3 inflammasome was maintained at the control level. Our previous study on LHBGRA43 showed that bioactive peptides released by this strain in fermented milk can modulate innate immunity by suppressing the production of pro-inflammatory cytokines such as IL-6 and TNFα (Strahinic et al., 2013). Moreover, the reduced expression of *Tlr4* and its downstream mediator *Myd88* suggests that the anti-inflammatory properties of the tested strain are mediated by improved intestinal barrier integrity induced by treatment with LHBGRA43 and the further reduction in endotoxin availability (Rocha et al., 2016). In addition, our results showed that LHBGRA43 can enhance the anti-inflammatory response to glucocorticoids, as nuclear translocation of the glucocorticoid receptor was increased in hepatocytes. The immunosuppressive effect of LHBGRA43 confirms that this probiotic strain has a beneficial effect not only in reversing liver fat deposition, but also in delaying more severe conditions such as liver damage and fibrosis.

Supplementation with probiotics is a proven strategy to restore intestinal homeostasis and reduce intestinal permeability by modulating the composition of the intestinal microbiota and the resulting production of bacterial metabolites (Latif et al., 2023). Although a high-fat diet was not sufficient to cause significant disruption of intestinal permeability, at least not through the mechanism investigated here, supplementation with LHBGRA43 significantly reduced the passage of the FITC-dextran marker across the intestinal barrier and increased ZO-1 protein expression compared to the high-fat diet alone, indicating a protective effect of the probiotic on intestinal permeability. In addition, we demonstrated that supplementation with LHBGRA43 had a positive effect on gut microbial diversity, as shown by increased alpha diversity indices. However, beta diversity analysis indicated that both the high-fat diet and LHBGRA43 treatment significantly changed the composition of the gut microbiota compared to the control group, and that the combined treatment further influenced microbiota composition compared to the high-fat diet alone. Overall, the frequency of the most common bacterial phyla in the LHBGRA43-treated animals was similar to that in the control group, regardless of the high-fat diet. In addition to increased microbial richness and diversity and the clear clustering of microbial profiles in the experimental groups, the observed remodeling of the gut microbiota composition was also characterized by a reduction in the *Firmicutes/Bacteroidota* (F/B) ratio. A lower F/B ratio has previously been associated with lower energy yield, improved metabolic parameters, and reduced liver fat accumulation (Tseng and Wu, 2019). This may be related to lower body mass and improved liver fat deposition after treatment with LHBGRA43. Furthermore, within the phylum *Bacteroidota*, the genus *Alistipes* was significantly increased after treatment with LHBGRA43. Although our findings refer to *Alistipes* at the genus level, certain *Alistipes* species, such as *Alistipes shahii*, have been shown to increase the expression of tight junction proteins (Lin et al., 2025). This is consistent with the observed increase in the tight junction protein ZO-1, which plays a crucial role in mucosal repair, especially after inflammatory conditions (Kuo et al., 2021). In line with this, treatment with LHBGRA43 also restored submucosal thickness to control levels, indicating a positive effect of the probiotic strain on the structural integrity of the gut epithelium. The absence of differences in villi length, crypt depth, or mucosa thickness may be due to dynamic intestinal remodeling under a high-fat diet. Early and prolonged exposure induces various structural changes that, along with physiological variability, can mask measurable differences at a single time point (Baldassano et al., 2013; Xie et al., 2020; Li X. et al., 2023).

Treatment with LHBGRA43 also led to an increase in the genera *Acetatifactor* and *Odoribacter*, which have previously been shown to contribute to the production of SCFAs (Portincasa et al., 2022). In our study, the decrease in propionic, butyric and hexanoic acid levels typical of HFD was not restored by supplementation with LHBGRA43. However, the shift in microbial composition induced by LHBGRA43 administration was accompanied by a decrease in heptanoic acid and 4-methylvaleric acid. The latter is a branched-chain fatty acid derived from microbial leucine fermentation (Wang et al., 2022). To date, there is no direct, proven correlation between heptanoic acid concentration and *Lactobacillus* treatment in relation to hepatic steatosis. However, studies have shown that branched-chain fatty acids decrease significantly when *Lactobacillus* probiotics are introduced, while SCFAs remain unchanged (Markowiak-Kopeć and Śliżewska, 2020; Asensio-Grau et al., 2023). The observed effect of LHBGRA43 on 4-methylvaleric acid can be attributed to several previously described properties of this strain (Strahinic et al., 2013). First, LHBGRA43 has been shown to exhibit strong antimicrobial activity against *Clostridium sporogenes* (Banina et al., 1998), a known producer of 4-methylvaleric acid. Second, its ability to rapidly acidify the environment during fermentation likely suppresses the growth of proteolytic bacteria responsible for amino acid degradation (Strahinić et al., 2013). Third, LHBGRA43 expresses a unique PrtH proteinase that enables efficient utilization of casein and β-lactoglobulin as nitrogen sources (Lozo et al., 2011), thereby reducing the availability of free leucine for microbial conversion to 4-methylvaleric acid. As elevated levels of 4-methylvaleric acid are associated with inflammation and impaired intestinal barrier function, it can be hypothesized that the mechanism behind the attenuation of gut-derived inflammatory signals by LHBGRA43 may lie in the reduced production of this particular metabolite.

Previous studies have reported clear sex differences in MASLD pathogenesis and responses to probiotics (Puhan et al., 2025). As this study included only male mice, the observed beneficial effects of the LHBGRA43 strain cannot be fully generalized to females. To address this limitation, future studies should determine whether these effects also occur in female mice and explore potential sex-specific mechanisms. Future research should also consider including a positive control. Since the current study was designed to investigate whether the probiotic strain exerts a beneficial effect in the MASLD model, comparison with established therapies is lacking. Subsequent comparative experiments with a positive control could provide a direct assessment of the relative efficacy of the treatment.

In summary, this study demonstrates that oral administration of the probiotic bacterial strain LHBGRA43 is a promising therapeutic approach to reduce hepatic steatosis. The beneficial effects of this strain begin in the gut by restoring gut microbiota composition and improving intestinal barrier integrity. Additional benefits include improved hepatic lipid metabolism, suppression of lipogenesis, reduction of lipotoxicity, and decreased inflammation. This immunosuppressive effect is most likely mediated by LHBGRA43’s ability to reduce the concentration of 4-methylvaleric acid. Reducing lipotoxicity and inflammation is crucial to prevent the progression of steatosis to more severe conditions such as steatohepatitis and fibrosis, which can lead to liver damage. Since the beneficial effects of LHBGRA43 have been observed under a high-fat diet, its supplementation is particularly advantageous for individuals who cannot permanently or significantly change their dietary habits but require additional treatment to prevent or reduce fat accumulation in the liver. A postmarketing clinical trial evaluating the efficacy of LHBGRA43 in individuals with MASLD is currently ongoing. Future research could explore the use of this strain as a complementary therapy. The probiotic may be combined with dietary and lifestyle interventions or other microbiota-targeted treatments to mitigate the progression of steatosis to more severe liver conditions. In addition, understanding the precise molecular mechanisms behind the hepatoprotective effects of LHBGRA43 may guide the development of next-generation probiotics and support the implementation of personalized therapeutic approaches for the treatment of fatty liver disease.

## Data Availability

The datasets presented in this study can be found in online repositories. The names of the repository/repositories and accession number(s) can be found below: Figshare repository, https://doi.org/10.6084/m9.figshare.29940587.v2.
