# Supplementary material for: Oral administration of the probiotic strain Lactobacillus helveticus BGRA43 reduces high-fat diet–induced hepatic steatosis in mice and restores gut microbiota composition
Source: Front Pharmacol. 2025 Nov 4;16:1688777. doi: 10.3389/fphar.2025.1688777 (PMC12623162; doi:10.3389/fphar.2025.1688777)
Supplement: Supplementary file 1 [file Table1.docx]

Supplementary Material

# Supplementary Figures and Tables

**Supplementary Table 1. GC/MS data for the SCFA standards.**

| No. | SCFA | Rt (min) | Chemical Formula | Mass | Major fragments, mass(intensity) |
| --- | --- | --- | --- | --- | --- |
| 1 | Acetic acid | 3.27 | C_2_H_4_O_2_ | 60 | 15(17), 29(8),43(100),45(90),60(75) |
| 2 | Propionic acid | 4.65 | C_3_H_6_O_2_ | 74 | 28(93), 29(83),45(90),57(47),73(65),74(100) |
| 3 | 2-Methyl Propionic acid | 5.15 | C_4_H_8_O_2_ | 88 | 27(15), 39(17),41(47)43(100),45(15),55(83),73(44),88(10) |
| 4 | Butyric acid | 5.78 | C_4_H_8_O_2_ | 88 | 27(14),41(16),55(6),60(100),73(32) |
| 5 | Valeric acid | 6.83 | C_5_H_10_O_2_ | 102 | 27(12),29(11),41(18),45(14),55(12),60(100),73(36),87(2) |
| 6 | 4‐Methylvaleric acid | 7.41 | C_6_H_12_O_2_ | 116 | 27(26),29(22),41(45),43(58),55(46),57(100),60(41),73(58),74(63),83(15),101(5) |
| 7 | Hexanoic acid | 7.72 | C_6_H_12_O_2_ | 116 | 27(12),29(10),41(20),43(16),55(10),60(100),73(45),87(13) |
| 8 | Heptanoic acid | 8.52 | C_7_H_14_O_2_ | 130 | 27(13),29(12),41(32),43(32),55(6),60(100),73(47),87(22),101(7) |

.
