# Supplementary material for: Oral administration of the probiotic strain Lactobacillus helveticus BGRA43 reduces high-fat diet–induced hepatic steatosis in mice and restores gut microbiota composition
Source: Front Pharmacol. 2025 Nov 4;16:1688777. doi: 10.3389/fphar.2025.1688777 (PMC12623162; doi:10.3389/fphar.2025.1688777)
Supplement: Supplementary file 2 [file DataSheet1.pdf]

Figure 1C

PageRuler Prestained Protein Ladder, 26616, ThermoScientific

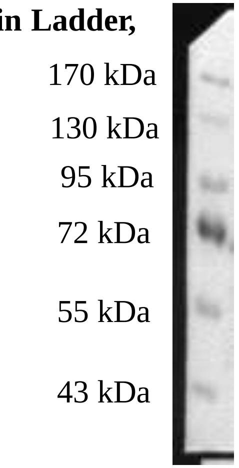

M12, 8 % gel, liver - nuclear fraction

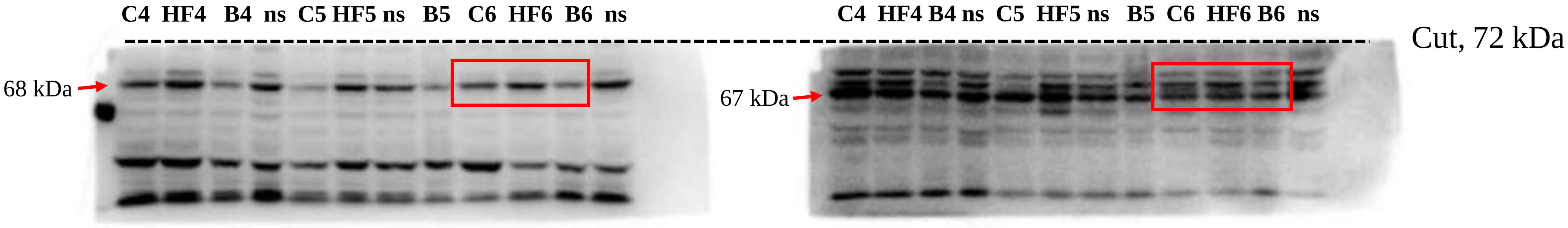

SREBP-1c

Lamin B1

Figure 1E

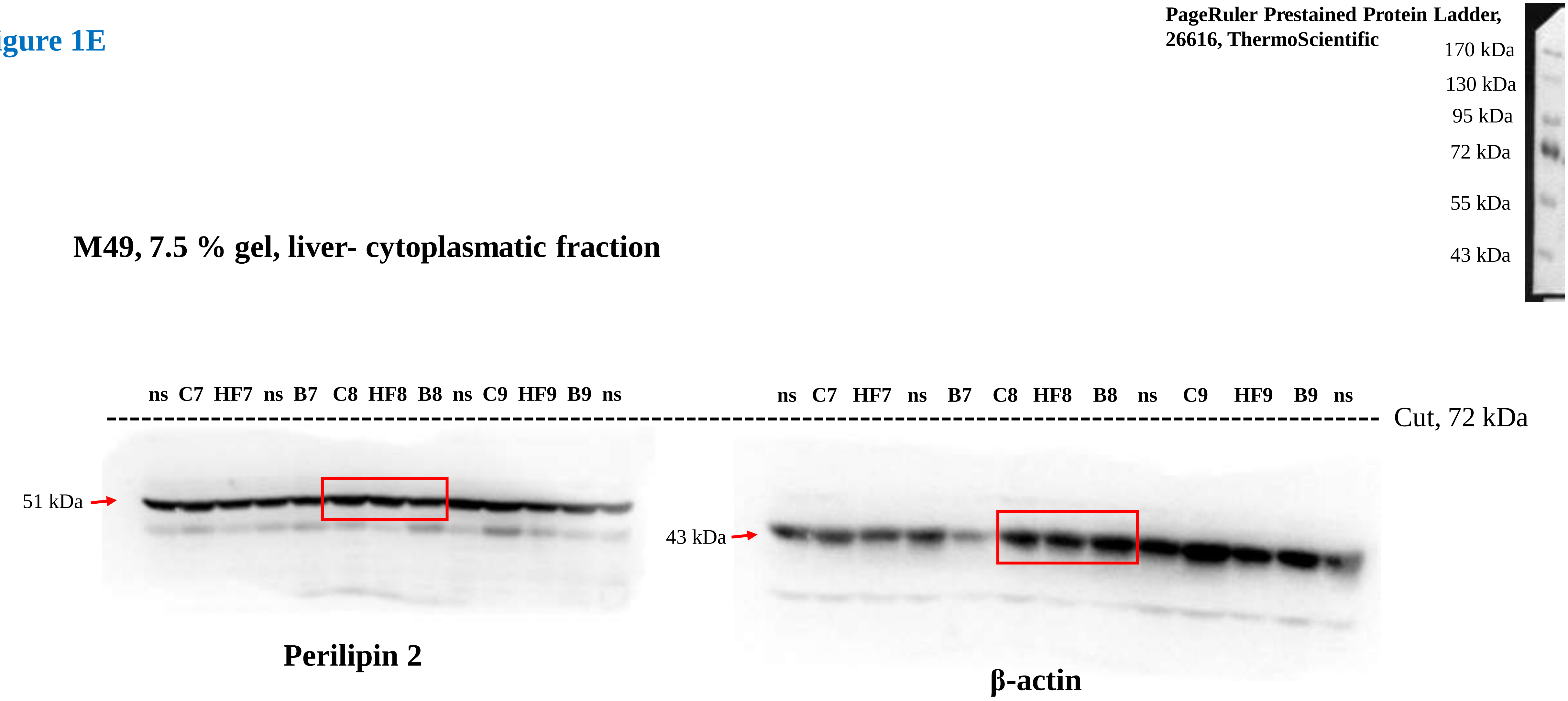
PageRuler Prestained Protein Ladder,
26616, ThermoScientific
170 kDa
130 kDa
95 kDa
72 kDa
55 kDa
43 kDa
M49, 7.5 % gel, liver- cytoplasmatic fraction
ns C7 HF7 ns B7 C8 HF8 B8 ns C9 HF9 B9 ns
ns C7 HF7 ns B7 C8 HF8 B8 ns C9 HF9 B9 ns
Cut, 72 kDa
51 kDa
43 kDa
Perilipin 2
β-actin

Figure 2B

M44, 7.5 % gel, liver - cytoplasmatic fraction

C4 HF4 B4 ns C5 HF5 ns B5 C6 HF6 B6 ns ns

NLRP3

100 kDa

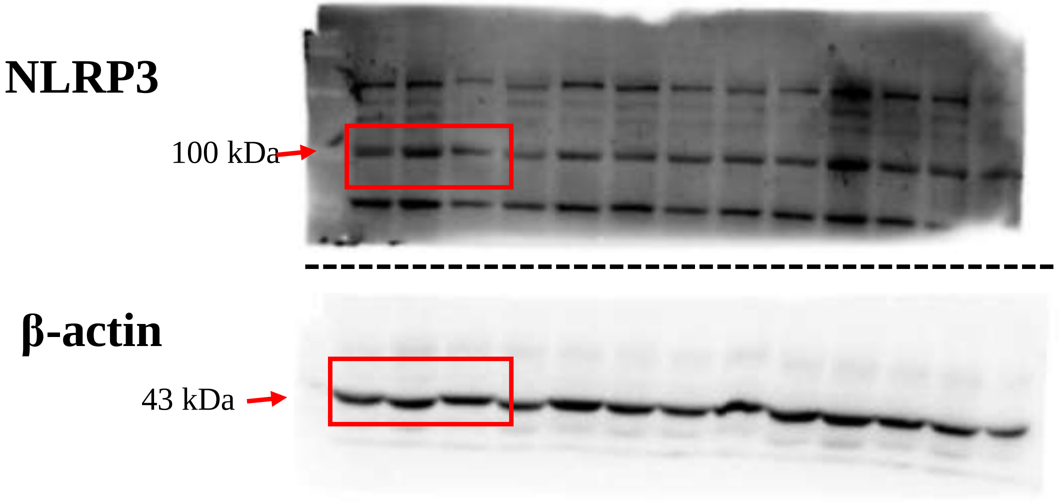

Cut, 55 kDa

β-actin

43 kDa

PageRuler Prestained Protein Ladder, 26616, ThermoScientific

170 kDa
130 kDa
95 kDa
72 kDa
55 kDa
43 kDa

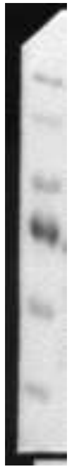

Figure 2C

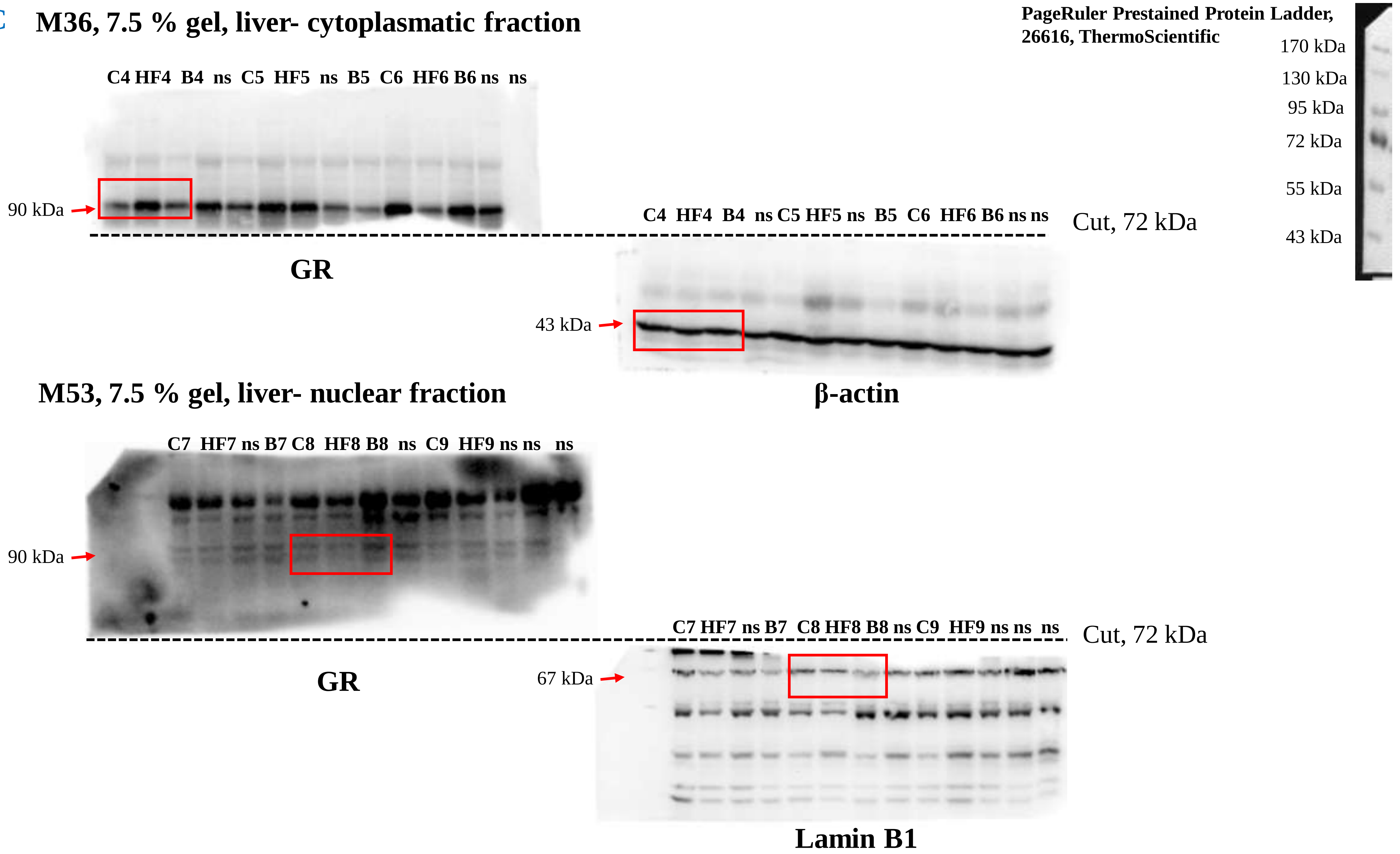
M36, 7.5 % gel, liver- cytoplasmatic fraction
PageRuler Prestained Protein Ladder, 26616, ThermoScientific
170 kDa
130 kDa
95 kDa
72 kDa
55 kDa
43 kDa
C4 HF4 B4 ns C5 HF5 ns B5 C6 HF6 B6 ns ns
90 kDa
GR
C4 HF4 B4 ns C5 HF5 ns B5 C6 HF6 B6 ns ns
Cut, 72 kDa
43 kDa
β-actin
M53, 7.5 % gel, liver- nuclear fraction
C7 HF7 ns B7 C8 HF8 B8 ns C9 HF9 ns ns ns
90 kDa
GR
C7 HF7 ns B7 C8 HF8 B8 ns C9 HF9 ns ns ns
Cut, 72 kDa
67 kDa
Lamin B1

Figure 3D

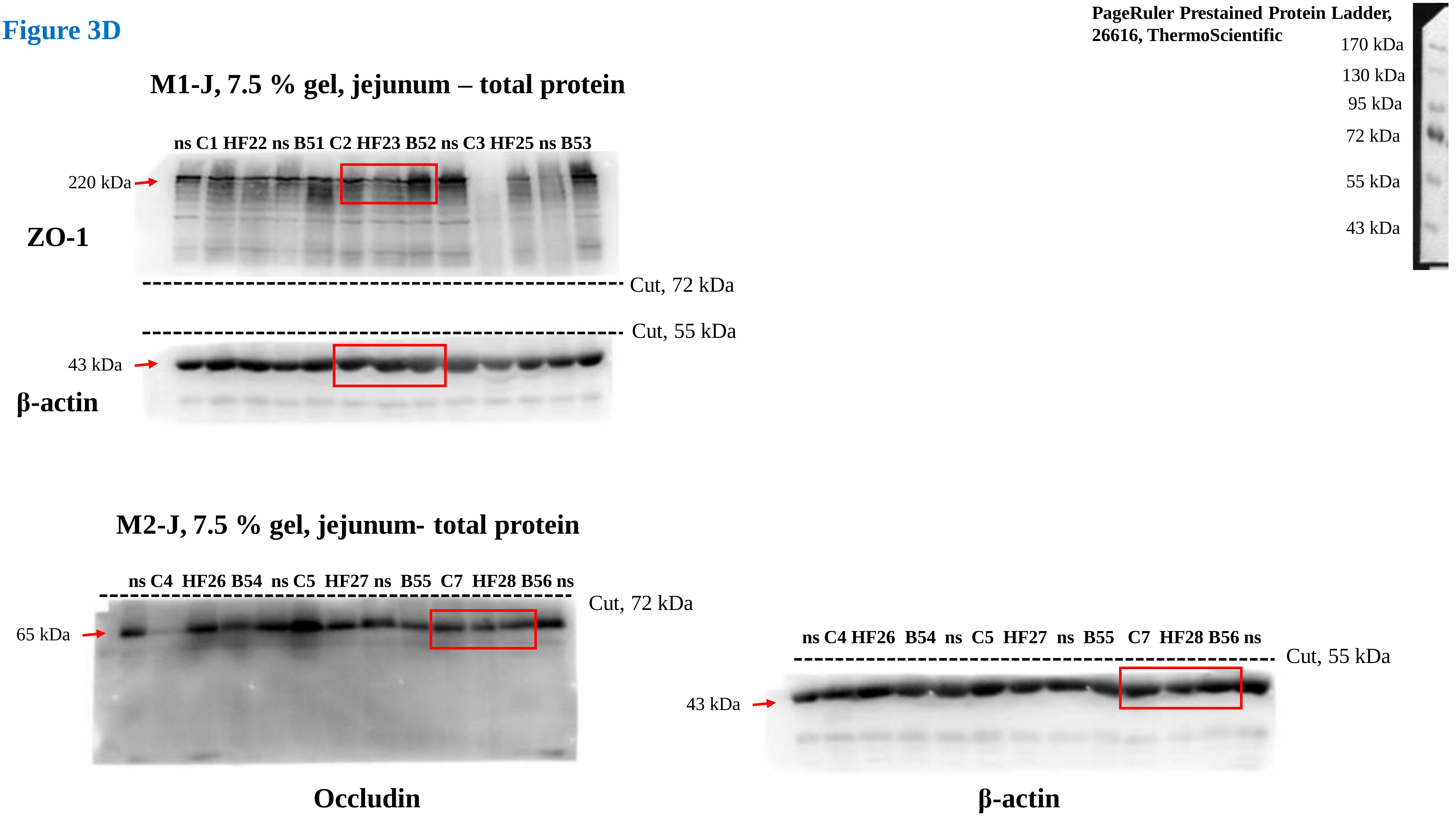

PageRuler Prestained Protein Ladder, 26616, ThermoScientific
170 kDa
130 kDa
95 kDa
72 kDa
55 kDa
43 kDa
M1-J, 7.5 % gel, jejunum – total protein
ns C1 HF22 ns B51 C2 HF23 B52 ns C3 HF25 ns B53
220 kDa
ZO-1
Cut, 72 kDa
Cut, 55 kDa
43 kDa
β-actin
M2-J, 7.5 % gel, jejunum- total protein
ns C4 HF26 B54 ns C5 HF27 ns B55 C7 HF28 B56 ns
Cut, 72 kDa
65 kDa
Occludin
ns C4 HF26 B54 ns C5 HF27 ns B55 C7 HF28 B56 ns
Cut, 55 kDa
43 kDa
β-actin
